# The Ratio of Height to Thyromental Distance (RHTMD) and Height to Sternomental Distance (RHSMD) as the Predictive Tests for Difficult Tracheal Intubation

**DOI:** 10.7759/cureus.28734

**Published:** 2022-09-03

**Authors:** Amruthraju CM, Sudhir S Rao, Rooparani K, Vinay R, Vikas KN, Deepak T S

**Affiliations:** 1 Department of Anaesthesiology, Dr. Anji Reddy Multispeciality Hospital, Guntur, IND; 2 Department of Anaesthesiology, MS Ramaiah Medical College, Bangalore, IND; 3 Department of Emergency Medicine, MS Ramaiah Medical College, Bangalore, IND

**Keywords:** ratio of height to thyromental distance, cormack and lehane, ratio of height to sternomental distance, thyromental distance, difficult intubation

## Abstract

Background: Pre-operative evaluation is a cornerstone in identifying patients with a risk of difficulty in intubation. Thyromental distance (TMD) is the most commonly used predictor of difficult intubation. However, it’s not a reliable indicator of difficulty during intubation because it differs with patients’ body & size proportion. The present study was done for the evaluation of the ratio of height to thyromental distance (RHTMD) and ratio of height to sternomental distance (RHSMD) as difficult airway predictors.

Methods: Data was taken from 400 consecutive patients posted for the need for anesthesia with intubation during surgery. Preoperatively examination of RHTMD and RHSMD was done. Difficulty during intubation has been explained in this current study with Cormack and Lehane grade 3 or 4. The positive and negative predictive values, as well as sensitivity and specificity of individual tests, were calculated as per the recognized formula.

Results: The study enrolled 400 patients, which include a maximum number of participants (138 [34.5%]) from the 41-50 year age group. On analyzing RHTMD and RHSMD, the former was found to have a better predictive value than RHSMD (p=0.001). RHTMD & RHSMD was found to have 62.5% & 37.50% sensitivity, respectively. RHTMD was found to have better specificity, positive & negative predictive values, and accuracy than RHSMD.

Conclusion: RHTMD was observed to have superior precision in anticipating difficulty in intubation compared to RHSMD.

## Introduction

Anticipating and managing difficult intubation is a vital skill in the practice of anaesthesiology. Difficult/failed intubation is one of the major causes of anesthesia-related morbidity and mortality. In patients with difficult airways, the majority of patients (85%) involve brain damage. 

An important reason for mortality as well as morbidity in subjects undergoing surgery with general anesthesia is failed airway management. The number of such incidents ranges from 1.5%-13% in individuals undergoing a surgical procedure, which leads to a significant amount of unfavorable outcomes during anesthesia practice [1-4]. So, evaluation before surgery is significant in identifying subjects who have difficult airways.

Numerous advances have been made to succeed in predicting unexpected difficult airways; the currently used tests such as Patil’s measurement of Thyromental distance (TMD), Mallampatti test, and Wilson scoring system have been proved in previous studies to have significant false positive rates, which makes their clinical use less significant [5-7].

The ratio of height to thyromental distance (RHTMD) and the ratio of height to sternomental distance (RHSMD) are taken as beneficial screening tests for the prediction of difficulty in laryngoscopy. An extensive literature search revealed that very few research studies had been conducted comparing the two. This study was done for the evaluation of the ratio of height to thyromental distance (RHTMD) and ratio of height to sternomental distance (RHSMD) as difficult airway predictors.

## Materials and methods

This is an observational study done in a teaching hospital with prior approval from the ethics and research committee. Four hundred patients with the American Society of Anesthesiologists physical status I or II, adult patients aged above 18 years planned for general anesthesia with endotracheal intubation, were chosen. Patients with midline neck swelling, mouth opening less than 3cm, known cardio-respiratory compromise, and pregnant patients were excluded.

After evaluating preoperatively, informed consent was taken in writing. Airway examination was performed by an experienced anaesthesiologist in all cases. The Modified Mallampatti (MMP) grade was measured in a sitting position with the patient's mouth wide open and protruding tongue without saying "Ah" [8]. TMD is taken from the thyroid notch to the inferior margin of the mentum with the mouth closed and the head held in the fully extended position. Sternomental distance (SMD, in cm) was measured from manubrium sterni to the mentum with head in extension position and mouth closed. The height of the patient was measured using the stadiometer consisting of a ruler and a sliding horizontal headpiece that can be fixed above the head to measure height. RHTMD was derived using the formula of the ratio of patient height in cm to TMD in cm. Similarly, the ratio of height and Sternomental distance was used for the calculation of RHSMD. RHTMD > 23.5, RHSMD > 12.5, Mallampati grade 3 and 4, and TMD < 6.0 cm, SMD < 12.5 cm were considered as difficult intubation predictors [8].

After pre-oxygenation of all patients, induction was done with an intravenous injection of propofol (1%) 2 mg/kg and paralyzed using intravenous succinylcholine 2 mg/kg for facilitation of laryngoscopy and intubation. Laryngoscopy was done with full relaxation, with appropriate positioning, and with a suitable blade size of 3 or 4 numbers by a senior consultant in the department. Visualization of the vocal cord was done by using a modified Cormack Lehane (C-L) classification [9]. Grade 3 and 4 were considered difficult laryngoscopy in Cormack and Lehane’s classification. Thereafter, an appropriate size endotracheal tube was secured in the trachea and confirmed by end-tidal CO2 concentration.

External laryngeal pressure was allowed before endotracheal intubation after evaluating the patient. However, the patient was excluded from the study. The preoperative assessment data and laryngoscope findings were used together to find the accuracy test of RHTMD in the prediction of difficult intubation. Sensitivity, specificity, and positive & negative predictive values among individual tests were derived using a standard formula. The sample size was calculated with the anticipated sensitivity and specificity of RHTMD at 93% and 62%, respectively, and a 95% confidence level [5]. IBM Corp. Released 2021. IBM SPSS Statistics for Windows, Version 28.0. Armonk, NY: IBM Corp was used for the analysis of the data. P value less than 0.05 was considered significant.

## Results

The study enrolled 400 patients, which include a maximum number of participants [138 (34.5%)] from the 41-50 year age group (Table 1). The study includes 209 males and 191 females, and all patients successfully completed the study. The mean body mass index of the patients was 23.7 ± 1.98 (Table 1).

**Table 1:** Demographic data ASA: American Society of Anesthesiologists; BMI: Body mass index

Variables		Number of patients (Percentage)
Age (years)	18-20	31 (7.75)
	21-30	65 (16.25)
	31-40	112 (28)
	41-50	138 (34.5)
	51-60	54 (13.5)
Gender	Male/Female	209 (52%)/ 191 (48%)
ASA Grade	I/II	212/188
BMI (kg/m^2^)	23.7 ± 1.98	

Table 2 depicts that 320 patients who predicted easy intubation on assessing RHTMD also had C-L grades of 1 or 2 (Table 2). However, 30 patients who were predicted as easy intubation on RHTMD had difficulty in performing the procedure (Table 2). Fifty patients who were anticipated as difficult intubation on measuring RHTMD also had C-L grade 3 or 4 (p=0.001) (Table 2). On assessing RHSMD, 318 & two patients were anticipated as easy and difficult intubation (Table 2). However, the anaesthesiologist observed 50 & 30 patients as difficult intubation (p=0.001) (Table 2).

**Table 2:** Distribution of predictive tests based on Cormack-Lehane grading C-L: Cormack Lehane grade; RHTMD: Ratio of height to thyromental distance; RHSMD: Ratio of height to sternomental distance *p<0.05

Tests		C-L grade			P value
		Easy (%)	Difficult (%)	Total (%)	
RHTMD	Easy	320 (100) (TN)	30 (37.5) (FN)	350 (87.5)	0.001*
	Difficult	0 (0) (FP)	50 (62.5) (TP)	50 (12.5)	
	Total	320 (80)	80 (20)	400 (100)	
RHSMD	Easy	318 (99.38) (TN)	50 (62.5) (FN)	368 (92)	0.001*
	Difficult	2 (0.62) (FP)	30 (37.5) (TP)	32 (8)	
	Total	320 (80)	80 (20)	400 (100)	

On analyzing RHTMD and RHSMD, the former was found to have a better predictive value than RHSMD (p=0.001) (Table 3). Out of a total of 344 patients with anticipated easy intubation by RHMTD, nine patients were predicted as difficult on estimating RHSMD. Moreover, out of 56 patients with anticipated difficult intubation by RHMTD, only 21 patients were predicted as difficult upon the estimation of RHSMD (p=0.001) (Table 3).

**Table 3:** Analysis of RHTMD and RHSMD RHTMD: Ratio of height to thyromental distance; RHSMD: Ratio of height to sternomental distance *p<0.05

RHTMD	RHSMD		Total (%)	P value
	Easy <12.5 (%)	Difficult >12.5 (%)		
Easy <23.5	335 (90.5)	9 (30)	344 (86)	0.001*
Difficult >23.5	35 (9.5)	21 (70)	56 (14)	
Total	370 (92.5)	30 (7.5)	400 (100)	

Table 4 depicts the sensitivity, specificity, positive & negative predictive values, and accuracy of RHTMD and RHSMD. RHTMD & RHSMD was found to have 62.5% & 37.50% sensitivity, respectively. RHTMD was found to have better specificity, positive & negative predictive values, and accuracy than RHSMD (Table 4).

**Table 4:** Comparison of RHTMD and RHSMD RHTMD: Ratio of height to thyromental distance; RHSMD: Ratio of height to sternomental distance

Parameters	RHTMD	RHSMD
Sensitivity	62.5 (50.96-73.08)	37.50 (26.92-49.04)
Specificity	100 (98.85 - 100.00)	99.38 (97.76-99.92)
Positive predictive value	100	93.75 (78.55-98.40)
Negative predictive value	91.43 (88.94-93.40)	86.41 (84.29-88.29)
Accuracy	92.50 (89.47-94.88)	87 (83.30-90.14)

## Discussion

The ratio of height to thyromental distance (RHTMD) and the ratio of height to sternomental distance (RHSMD) are considered useful screening tests for predicting difficult laryngoscopy; however, very few studies exist comparing these two predictive tests [10]. Shiga T et al. performed a study on predicting the difficulty in intubation among routine subjects: a meta-analysis of bedside tests. Thirty-five studies, including 50,760 subjects, were taken from databases. This study demonstrated that a combination of MP and TMD was found to be very useful [11]. Mukesh Tripathi et al., in their study, predicted that short TMD; < 5 cm was associated with difficulty in endotracheal intubation in most of the patients. Then they described the small numbered Macintosh blade (No. 2) that could help in cases of difficulty during direct laryngoscopic intubations in short TMD adults than the Macintosh blade (No. 3) [12].

Wilson et al. observed 20 parameters for predicting difficulty during intubation. They found out that five of them (weight, jaw movement, receding mandible, head and neck movement, and buck teeth) were the main predictors of difficulty during intubation. The scoring system was introduced by taking into account all these parameters. The important effect of the observation was it would not be possible to recognize the difficulty in intubation of adult patients without having a high false positive rate [13].

As observed in previous studies, no single test can be taken as a guarantee in predicting difficulty during intubation. There is a definite requirement for a fast and easily performable test. RHTMD introduced individual proportions to the concept of TMD measurements taken for females with a height measuring 160 cm and a 190 cm measurement in males can have different jaw ratios corresponding with the structures surrounding [14,15].

Results, as discussed above, clearly have demonstrated that RHTMD has the highest predictive value in comparison with RHSMD. Our study estimated the incidence of difficulty during intubation as 3.3%. The data also showed that RHMTD was found to be more sensitive and specific as compared to RHSMD. Moreover, the accuracy of RHMTD was observed to be much better than RHSMD (Table 4). 

Farzi et al. conducted a study on adult patients to compare airway tests RHTMD, RHSMD, and assessment of oropharyngeal view by MMC [16]. The study demonstrated that RHSMD had the least false negative value, a cut-off point of RHSMD > 12.5 was not different between men and women, and RHSMD ≥ 12.5 had a direct relationship with difficult laryngoscopy. In our study, RHSMD had good specificity and accuracy with a cut-off of 87.00%. Kumar S et al. also observed that RHTMD is more sensitive and specific compared to RHSMD [17]. They also supported that using RHTMD in combination with RHSMD increases the chance of predicting difficult intubation rather than using them alone, and both predictive tests were found to be highly statistically significant [17].

Among 320 intubations which were easy according to Cormack-Lehane grading, RHTMD and RHSMD predicted 320 and 318 intubations, respectively. Therefore, RHTMD (100%) is highly specific in predicting difficulty in intubation compared to RHSMD (99.38%). Out of 80 difficult intubations, 50 & 30 were predicted as same by RHTMD & RHSMD, respectively. Therefore, the positive predictive value of RHTMD & RHSMD was 91.43 & 86.41, respectively.

One of the limitations of the study was that the predictability of RHMTD and RHSMD as difficult airway parameters was not correlated with Modified Mallampatti Classification. Moreover, it would be much better if other parameters (upper lip bite test) of difficult airway assessment were used for correlating the predictability of RHMTD and RHSMD as difficult airway parameters.

## Conclusions

The study demonstrates the significance of the new, very handy & excellent bedside test, which involved the assessment of landmarks of anatomical significance to predict difficulty in intubation with direct laryngoscopy. This test was done on an acceptable number of adult patients and a wide age difference. RHTMD was found to have high specificity (100%) and sensitivity (62.5%), with an accuracy of 92.5% in the assessment of difficulty in intubation. RHTMD is highly specific & sensitive as compared to RHSMD. However, the combination of RHTMD & RHSMD can be used for a much better prediction of difficult intubation.
